# Identification of tryptophan metabolic gene-related subtypes, development of prognostic models, and characterization of tumor microenvironment infiltration in gliomas

**DOI:** 10.3389/fnmol.2022.1037835

**Published:** 2022-11-04

**Authors:** Yi Liu, Juan Xiang, Yiwei Liao, Gang Peng, Chenfu Shen

**Affiliations:** ^1^Department of Neurosurgery, Xiangya Hospital, Central South University, Changsha, Hunan, China; ^2^Department of Geriatrics, Xiangya Hospital, Central South University, Changsha, Hunan, China; ^3^National Clinical Research Center for Geriatric Disorders, Xiangya Hospital, Central South University, Changsha, Hunan, China

**Keywords:** tryptophan metabolic gene, tumor microenvironment, genomic variation, immunotherapy, temozolomide

## Abstract

**Background:**

Epigenetic regulation and immunotherapy of tumor microenvironment (TME) is a hot topic in recent years. However, the potential value of tryptophan metabolism genes in regulating TME and immunotherapy is still unclear.

**Materials and methods:**

A comprehensive study of glioma patients was carried out based on 40 tryptophan metabolic genes. Subsequently, these prognostic tryptophan metabolic genes are systematically associated with immunological characteristics and immunotherapy. A risk score model was constructed and verified in the Cancer Genome Atlas (TCGA) and the Chinese Glioma Genome Atlas (CGGA) cohorts to provide guidance for prognosis prediction and immunotherapy of glioma patients.

**Results:**

We described the changes of tryptophan metabolism genes in 966 glioma samples from genetic and transcriptional fields and evaluated their expression patterns from two independent data sets. We identified two different molecular subtypes and found that two subtypes were associated with clinicopathological features, prognosis, TME cell infiltration, and immune checkpoint blockers (ICBs). Then, four genes (IL4I1, CYP1A1, OGDHL, and ASMT) were screened out by univariate and multivariate cox regression analysis of tryptophan metabolism genes, and a risk score model for predicting the overall survival (OS) of glioma patients was constructed. And its predictive ability is verified using the CGGA database. At the same time, we verified the expression of IL4I1, CYP1A1, OGDHL, and ASMT four genes in glioma specimens and cell lines in GES4260 and GES15824. Therefore, we constructed a nomogram to improve the clinical applicability of the risk assessment model. The high risk score group, characterized by increased TMB and immune cell infiltration, was also sensitive to temozolomide immunotherapy. Our comprehensive analysis of tryptophan metabolic genes in gliomas shows that they play a potential role in tumor immune stromal microenvironment, clinicopathological features, and prognosis.

**Conclusion:**

Tryptophan metabolism genes play an indispensable role in the complexity, diversity, and prognosis of TME. This risk score model based on tryptophan metabolism gene is a new predictor of clinical prognosis and immunotherapy response of glioma, and guides a more appropriate immunotherapy strategy for glioma patients.

## Introduction

Glioma is the most common primary intracranial tumor of the central nervous system, of which glioblastoma is the most malignant and lethal subtype (Weller et al., 2015; Zhang et al., 2019; Jiang et al., 2021). Despite significant progress in standard treatments, including surgical resection, targeted radiotherapy, and chemotherapy (Aldape et al., 2019), the median survival time for people with glioblastoma, regardless of treatment, is 14–15 months (Ostrom et al., 2020). The field of immunotherapy and tumor electric field therapy (TTFields; Stupp et al., 2017; Mun et al., 2018; Sampson et al., 2020) has developed rapidly in recent years, but no substantial breakthroughs have been made. An increasing number of studies have analyzed tumor cell metabolomics to explore malignant phenotypes of gliomas and identify novel therapeutic targets (Ahmed and Chinnaiyan, 2014; Bi et al., 2020).

The metabolism of tryptophan, an essential amino acid, is considered an important endogenous metabolic feedback loop, helping to regulate the extent, duration, and cellular composition of immune responses. Tryptophan metabolism in cancer cells and/or cancer-associated stromal cells contributes to the inhibition of the anti-tumor immune response (Platten et al., 2021). Tryptophan metabolites can regulate not only cancer cells, but also the whole tumor microenvironment, and can promote immunosuppression and drug resistance (Fallarino et al., 2003; Greene et al., 2019; Li and Zhao, 2021). In addition, tryptophan metabolism, as a new immune checkpoint, can affect the immunotherapeutic outcome (Li et al., 2019). These observations led to the development of novel therapeutic agents with the aim of regulating or inhibiting tryptophan metabolism. The first molecular target for small molecular inhibitors was indoleamine-2,3-dioxygenase-1 (IDO1), which catalyzes the rate-limited conversion of tryptophan to canine uric acid. Indoleamine-2,3-dioxygenase-2 (IDO2) can also be therapeutically targeted to catalyze the conversion of tryptophan to canine urine; this target is upregulated in gliomas and other types of cancer (Mondanelli et al., 2021). However, these molecular therapeutic agents are still in clinical development, and other novel drug targets should still be explored.

The tumor microenvironment (TME) is known to influence the occurrence and development of tumors (Hinshaw and Shevde, 2019). In addition to tumor cells, the TME also includes fibroblasts, endothelial cells, immune cells, inflammatory cells, extracellular matrix elements, and diffusible cytokines and chemokines, which are secreted from tumor and stromal cells. Crosstalk between cancer cells and proximal immune cells produces the TME, which affects the development and progression of cancer (Pottier et al., 2015). Malignant cells interact with the surrounding cells *via* the circulatory and lymphatic systems to promote tumor angiogenesis and induce immune tolerance by releasing cellular signal molecules. The TME can also affect tumor progression, and tumor-infiltrating immune cells (TIIC) in the TME can be used to predict the prognosis of cancer (Lee and Cheah, 2019). Moreover, previous studies have found that TME plays a key regulatory role in glioma progression (Quail and Joyce, 2017). Highly complex tryptophan metabolic pathways exist not only in tumor cells, but also in the TME, and cancer cells are able to use the resultant metabolites to regulate tumor immune cell infiltration, resulting in immune resistance and immune escape (Vitale et al., 2019). Until now, most studies have only evaluated the influence of one or two tryptophan metabolic genes on gliomas; however, tumor-promoting effects likely result from the interaction of many genes in a highly coordinated manner. Therefore, a comprehensive understanding of how TME cell infiltration characteristics are mediated by multiple tryptophan metabolic genes will enable a more in-depth understanding of glioma biological mechanisms and better prediction of the efficacy of immunotherapy.

This study therefore aimed to comprehensively evaluate the expression profile of tryptophan metabolic genes in relation to the composition of the immune cell population in the TME and overall survival in people with glioma.

## Materials and methods

### Acquisition and preprocessing of datasets from patients with glioma

We downloaded RNA expression data for normal brain tissue from the Genotype–Tissue Expression (GTEx) dataset using the UCSC Xena online browser tool.^1^ RNA transcriptome, somatic mutation, and copy number data for glioma samples with matched clinical data from people with glioma were downloaded from the Cancer Genome Atlas (TCGA; https://portal.gdc.cancer.gov/), the Chinese Glioma Genome Atlas (CGGA; http://www.cgga.org.cn/), and Gene Expression Omnibus of NCBI (GEO; http://www.ncbi.nlm.hih.gov/gds). After excluding individuals with incomplete clinical data, 966 glioma specimens (TCGA, *n* = 662; CGGA, *n* = 304) were included in further analyses. The GSE4290 dataset containing 23 normal samples and 157 glioma samples was used to verify marker gene expression. The GSE15824 dataset contains NHA, LN018, LN215, LN229, LN319, and BS149 cell lines for validation of marker gene expression. Transcripts per kilobase million (TPM) values and RNA sequencing data (RSEM) values were converted and log2-normalized in order to compare gene expression profiles from different platforms. The limma package in R was used to analyze mRNA expression for tryptophan metabolic genes in glioma and normal brain tissue samples.

Clinical information for all 966 patients with glioma included in this study is shown in Supplementary Table S1. Clinical variables included age, gender, grade, follow-up time, and survival status. In addition, somatic mutation data and genetic frequency were analyzed using the maftools package in R (in the mutation annotation format).

### Consensus cluster analysis of tryptophan metabolic genes

Forty tryptophan metabolic genes (TRYPTOPHANMETABOLISM_PYROPTOSIS)^2^ were queried using the MSigDB website. The ConensusClusterPlus package in R was used for consistent unsupervised cluster analysis, in which patients were divided into different molecular subtypes according to expression levels of tryptophan metabolic genes. Clustering was based on the following criteria: a gradual and smooth increase was observed in the cumulative distribution function (CDF) curve; no groups had small sample sizes; intra-group correlation increased and inter-group correlation decreased after clustering.

### Relationship between subtypes and clinical features and prognosis of brain gliomas

To test the clinical value of the two subtypes determined by consensus clustering, we compared the relationships among molecular subtypes, clinicopathological features, and prognosis. The characteristics of the patients included age, gender, and tumor grade. Kaplan–Meier curves were generated using the survival and survminer packages in R to assess differences in overall survival (OS) among patients with different subtypes.

### Correlation between subtypes of gliomas and TME, immune cells, and immune checkpoint blockers

According to the expression profiles of glioma patients, the ESTIMATE algorithm was used to evaluate the abundance of immune cells and stromal cells (Yoshihara et al., 2013). The ESTIMATE algorithm produced three types of scores: positive reflection of stromal cell abundance (stromal score), positive reflection of immune cell abundance (immune score), and positive reflection of non-tumor components (ESTIMATE score). In addition, the CIBERSORT algorithm was used to calculate the proportion of 22 human immune cell subsets in each glioma sample (Newman et al., 2015). Samples with *p* < 0.05 were selected for further analysis, and the difference in abundance of immune cells among different subtypes was compared using a Wilcoxon rank sum test. We also analyzed the correlation between the two subtypes of different ICBs (PDCD1, CD274, PDCDLG2, CTLA4, CD80, and CD86).

### Construction and verification of gene prediction model for tryptophan metabolism

Nine hundred and sixty-six patients with glioma were divided into a training set (TCGA, n = 662) and verification set (CGGA, n = 304). In the training set, we used the survival package in R to screen these tryptophan metabolism genes by univariate Cox analysis to identify those related to glioma prognosis. Then, the genes that were most related to glioma prognosis were screened using multivariate Cox analysis of prognosis-related genes and survival rates. We then constructed a tryptophan metabolic gene prediction model that included prognosis-related genes, weighted with multivariate Cox regression coefficients according to the equation


$$\mathrm{Riskscore}=\Sigma \left({\mathrm{Expi}}^{*}\mathrm{Coefi}\right)$$

where Coefi and Expi represent the risk coefficient and expression of each gene, respectively. According to the median risk score, 662 patients in the training group were stratified into low risk (risk score < median) and high risk (risk score > median) groups for Kaplan–Meier survival analysis. Then, principal component analysis (PCA) was carried out using the ggplot2 package in R. The verification set was stratified in the same way. In each cohort, Kaplan–Meier analysis was used to evaluate the importance of prediction between high and low risk groups, and 1, 3, and 5-year receiver operating characteristic (ROC) curves were drawn further test the efficiency of prediction. Values for the area under the ROC curve (AUC) were calculated using the survival, survminer, and survivalROC packages in R.

### Evaluation of immune status, tumor mutation burden, and ICBs between high and low risk groups

CIBERSORT was used to quantify the abundance of 22 TIICs in heterogeneous samples from low and high risk groups. We also investigated the relationship between TIIC composition and risk score. We used box diagrams to examine differential expression levels between low and high risk groups and the different immune checkpoint genes (*PDCD1*, *CD274*, *PDCDLG2*, *CTLA4*, *CD80*, and *CD86*). In addition, we also analyzed the relationship between risk level and TMB.

### Response to temozolomide

Temozolomide (TMZ) is a first-line chemotherapeutic drug for patients with glioma. We predicted the chemotherapeutic response for each sample from the TCGA dataset based on the largest publicly available pharmacogenomics database [Genomics of Drug Sensitivity in Cancer (GDSC), https://www.cancerrxgene.org/]. The prediction process was implemented in R using the pRRophetic package, where the half maximum inhibitory concentration (IC50) of the sample was estimated using ridge regression, all parameters were set at default values, the combat correction for batch effect was used, and the tissue type was set to “all,” and repeated gene expression was summarized as the average value.

### Establishment and verification of a nomogram scoring system

Based on the results of our independent prognostic analysis, a predictive nomogram was developed using the RMS package in R according to the clinical features and risk score. In the nomogram scoring system, each variable has a score, and the sum of all scores for all variables in each sample are added to calculate a total score. The nomogram was evaluated using 1, 3, and 5-year survival times. The calibration chart for the nomogram was used to describe the difference between predicted 5-year survival events and virtual observations.

### Statistical analysis

All statistical analyses were performed in R version 4.1.2. Values with associated *p* < 0.05 were considered statistically significant.

## Results

### Tryptophan metabolic gene expression and its alteration in glioma

**Figure 1:**
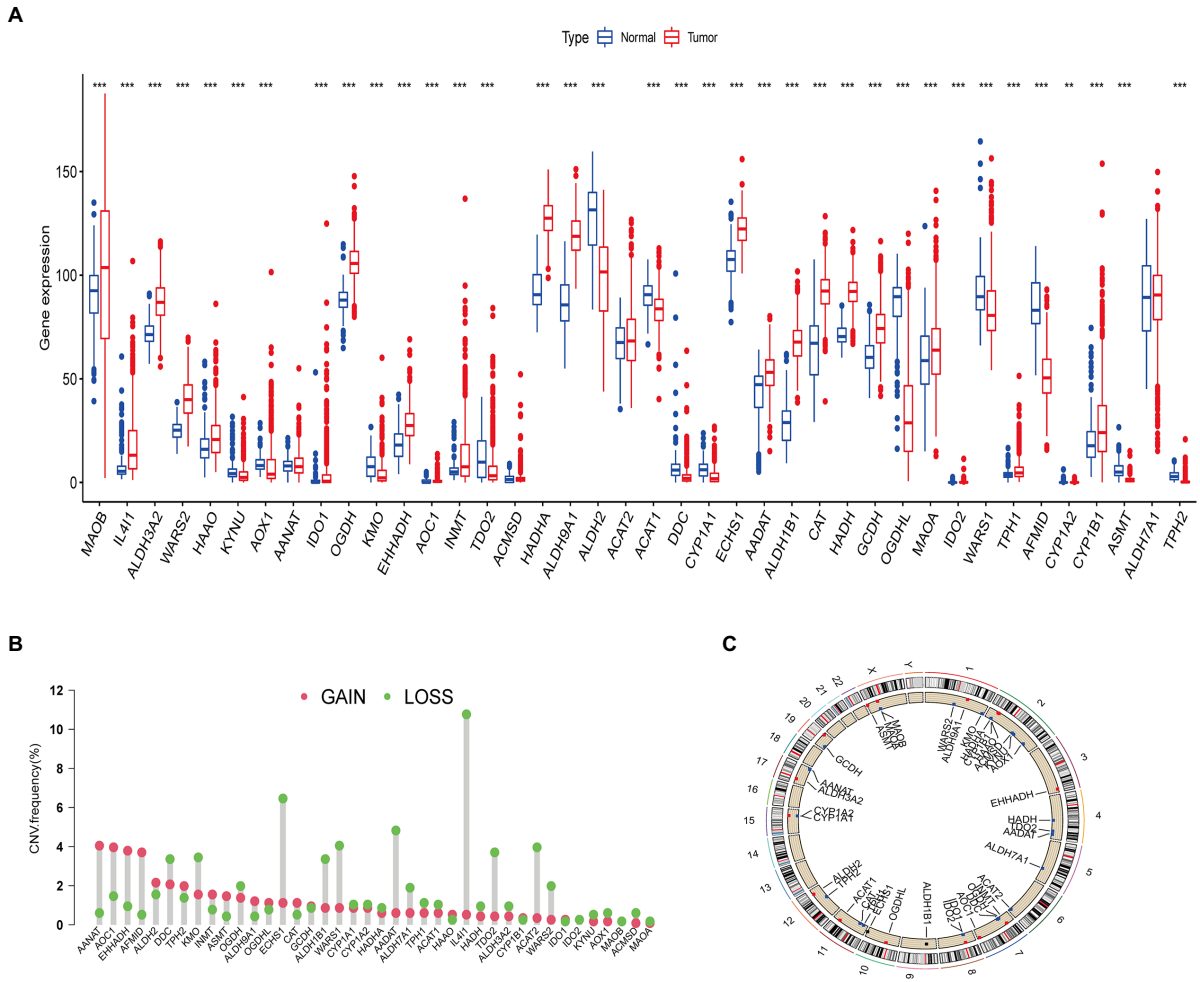
Genetic and transcriptional alterations in tryptophan metabolic genes in glioma. **(A)** Expression distributions of 40 tryptophan metabolic genes in normal vs. glioma tissues. **(B)** Frequencies of copy number variations (CNVs) including gain and loss, and non-CNV genetic variants among tryptophan metabolic genes. **(C)** Locations of CNVs in tryptophan metabolic genes across the 23 human chromosomes. *p < 0.05, **p < 0.01, ***p < 0.001.

First, we analyzed the differential expression of 40 tryptophan metabolic genes using the TCGA and GTEx databases (Figure 1A). There was a significant difference in gene expression pattern between the glioma and normal groups. The expression of *MAOB*, *IL4I1*, *ALDH3A2*, *WARS2*, *HAAO*, *AANAT*, *IDO1*, *OGDH*, *EHHADH*, *AOC1*, *INMT*, *HADHA*, *ALDH9A1*, *ALDH2*, *ECHS1*, *AADAT*, *ALDH1B1*, *CAT*, *HADH*, *GCDH*, *MAOA*, *IDO2*, *TPH1*, and *CYP1B1* was higher in glioma than normal brain tissue samples. Conversely, the expression of *KYNU*, *AOX1, KMO*, *TDO2*, *ACAT1*, *DDC*, *CYPIAI*, *OGDHL*, *WASRS1*, *AFMID*, *CYP1A2*, *ASMT*, and *TPH2* was lower in glioma than normal brain tissue samples. In addition, copy number variations (CNVs) were common in all 40 tryptophan metabolic genes. Among them, *AANAT*, *AOC1*, *EHHADH*, and *AFMID* had an increased copy number, while *ECHS1*, *IL4I1*, and *AADAT* showed a deletion of copy number (Figure 1B). Figure 1C shows the location of the CNV changes in the genes on their respective chromosomes. In addition, somatic mutation rates for all 40 genes were summarized and analyzed; the somatic mutation rates of tryptophan metabolism genes in the TCGA-GBM and GBM cohorts were 9.23% and 4.94%, respectively (Supplementary Figure S1).

The results of our analysis show that there are significant differences in the expression level and copy number of tryptophan metabolic genes between glioma and normal brain tissue samples, indicating that these genes potentially play a role in the occurrence of glioma.

### Identification of tryptophan metabolic gene subtypes in gliomas

**Figure 2:**
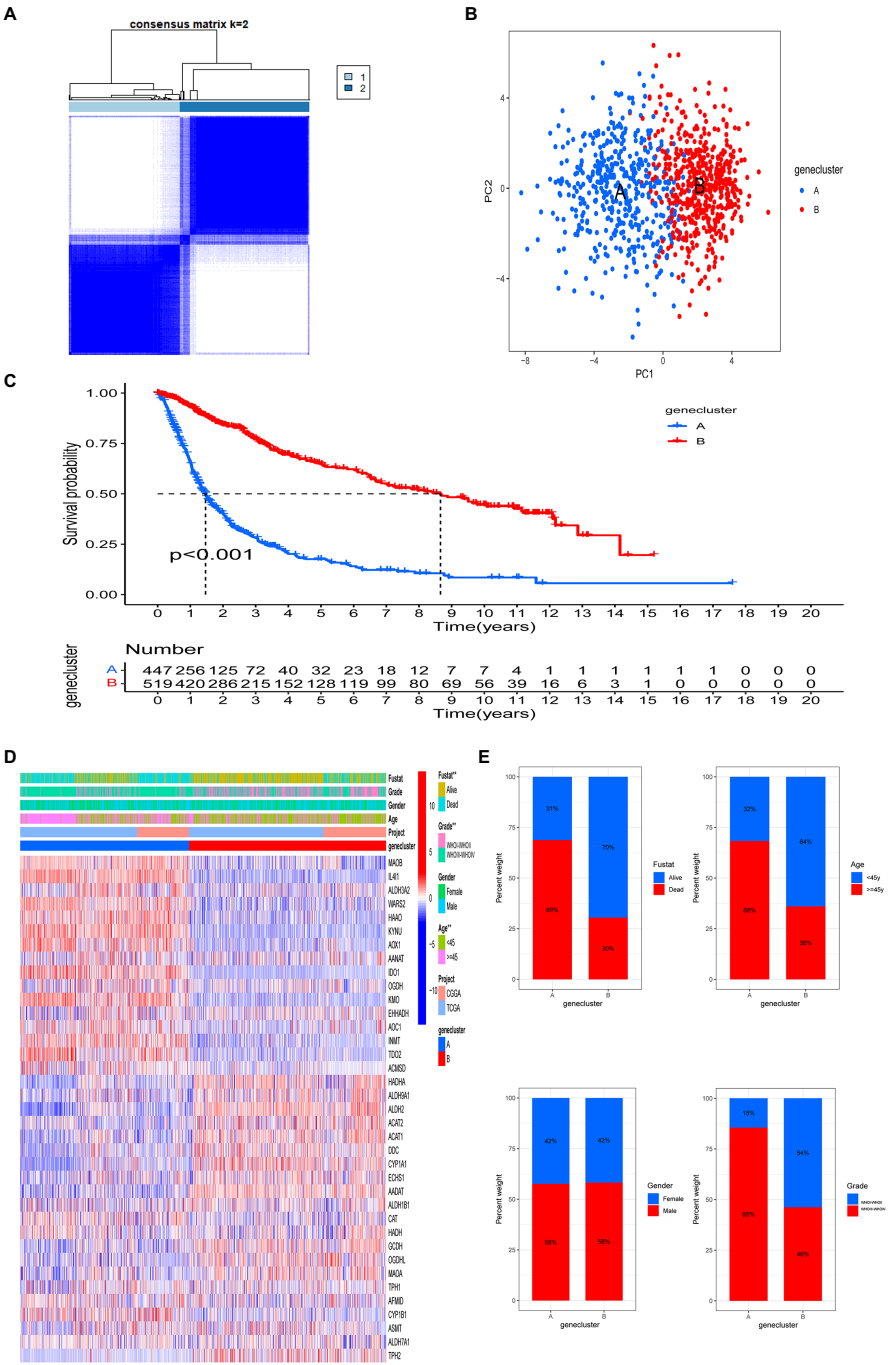
Two distinct tryptophan metabolic gene expression subtypes and the clinicopathological and biological characteristics of these subtypes, stratified by consistent clustering. **(A)** Consensus matrix heatmap defining two clusters (*k* = 2) and areas of correlation. **(B)** Principal component analysis showing a notable difference in transcriptomes between the two subtypes. **(C)** Univariate analysis showing genetic subtypes related to overall survival (OS). **(D)** Differences in clinicopathologic features and tryptophan metabolic gene expression levels between the two subtypes. **(E)** Comparisons of OS status, gender, WHO tumor stage, and age between two genetic subtypes. Significance: **p < 0.01.

We used a consensus clustering algorithm to classify patients with gliomas according to the expression profiles of the 40 tryptophan metabolic genes included in our study (Supplementary Figure S2). Our results show that k = 2 seemed to be the best choice, stratifying the entire cohort into subtype A (*n* = 447, 46.3%) and subtype B (*n* = 519, 53.7%; Figure 2A). PCA showed that there were significant differences in tryptophan metabolic gene transcription profiles between the two clusters (Figure 2B). The Kaplan–Meier curve showed that subtype B had a significant advantage in OS over subtype A (Figure 2C). In addition, in a comparison of the clinicopathological features of different glioma subtypes, we found significant differences in tryptophan metabolic gene expression and clinicopathology between the two glioma clusters (Figure 2D). The proportion of individuals who died were significantly higher in subtype A (69%) than subtype B (30%); compared with subtype B, patients in subtype A were also significantly older at diagnosis (68 vs. 36% aged 45 or older), with a higher tumor grade (85 vs. 46%; Figure 2E).

### Immunological characteristics of subtypes A and B

**Figure 3:**
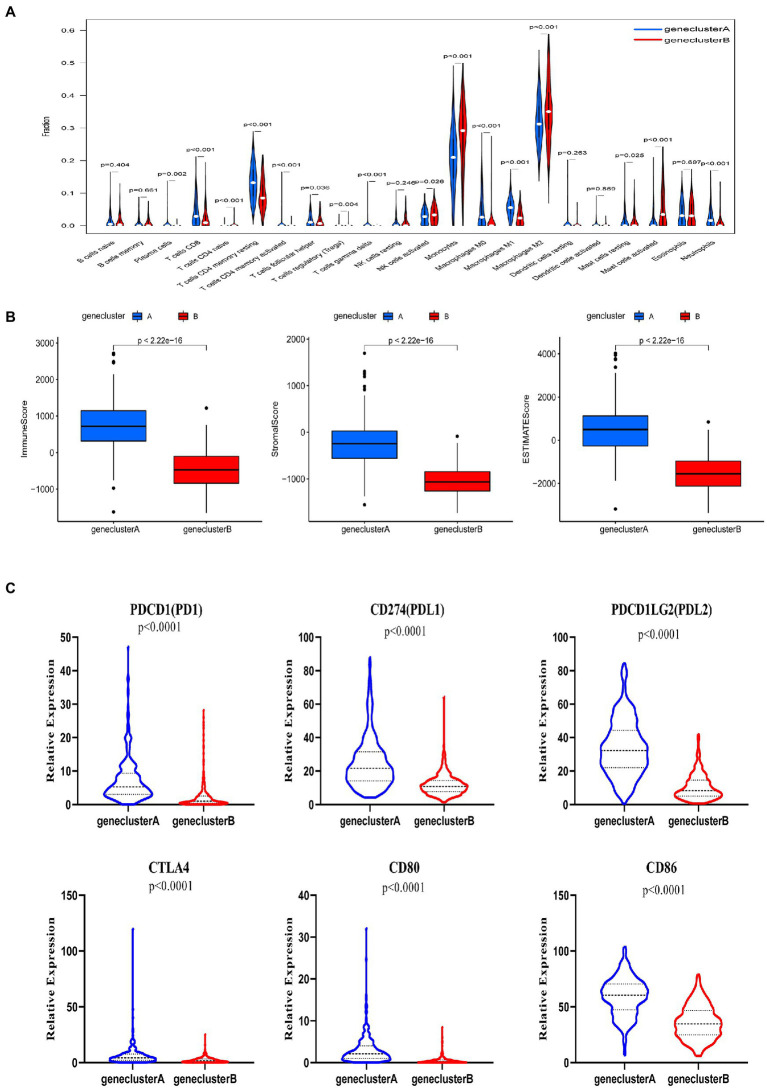
Correlations between tumor immune cell microenvironments and two glioma subtypes, defined by differential expression of tryptophan metabolic genes. **(A)** Abundance of 22 infiltrating immune cell types between the two glioma subtypes. **(B)** Correlations between the two glioma subtypes and tumor microenvironment score (immune score, stromal score, and ESTIMATE score). **(C)** Expression levels of *PD-1*, *PD-L1*, *PDL2*, *CTLA4*, *CD80*, and *CD86* between the two glioma subtypes.

We used the CIBERSORT algorithm to evaluate the correlation between subtype and immune cell composition in each glioma sample. There was a significant difference in the proportion of most immune cell types between the two subtypes (Figure 3A). The infiltration levels of plasma cells, CD8^+^ T cells, activated CD4^+^ memory T cells, resting CD4^+^ memory T cells, follicular helper T cells, regulatory T cells, γ δ T cells, M0 and M1 macrophages, and resting mast cells were significantly higher in subtype A than subtype B. Conversely, the infiltration levels of immature CD4^+^ T cells, activated natural killer (NK) cells, monocytes, M2 macrophages, activated mast cells, and neutrophils were significantly higher in subtype B than subtype A. We also evaluated TME scores for the two subtypes (interstitial score, immune score, and ESTIMATE score) using the ESTIMATE R package. Briefly, a higher interstitial score or immune score represents a higher relative content of stromal or immune cells in the TME, while the ESTIMATE score is an aggregation of the TME intermediate and immune scores. The TME score for individuals in subtype A was higher than those in subtype B (Figure 3B). These results together suggest that subtype A is associated with a higher level of TIIC infiltration, which may be useful for immunotherapy. In addition, the expression of *PD1* and *CTLA* and their ligands (*PD-L1*, *PD-L2*, *CD80*, and *CD86*, all *p* < 0.001) in subtype A was significantly higher than that in subtype B (Figure 3C). These results showed that patients with gliomas in subtype A were more likely to have immune resistance, resulting in a worse prognosis.

### Identification and verification of a prognostic model based on tryptophan metabolic gene expression

**Figure 4:**
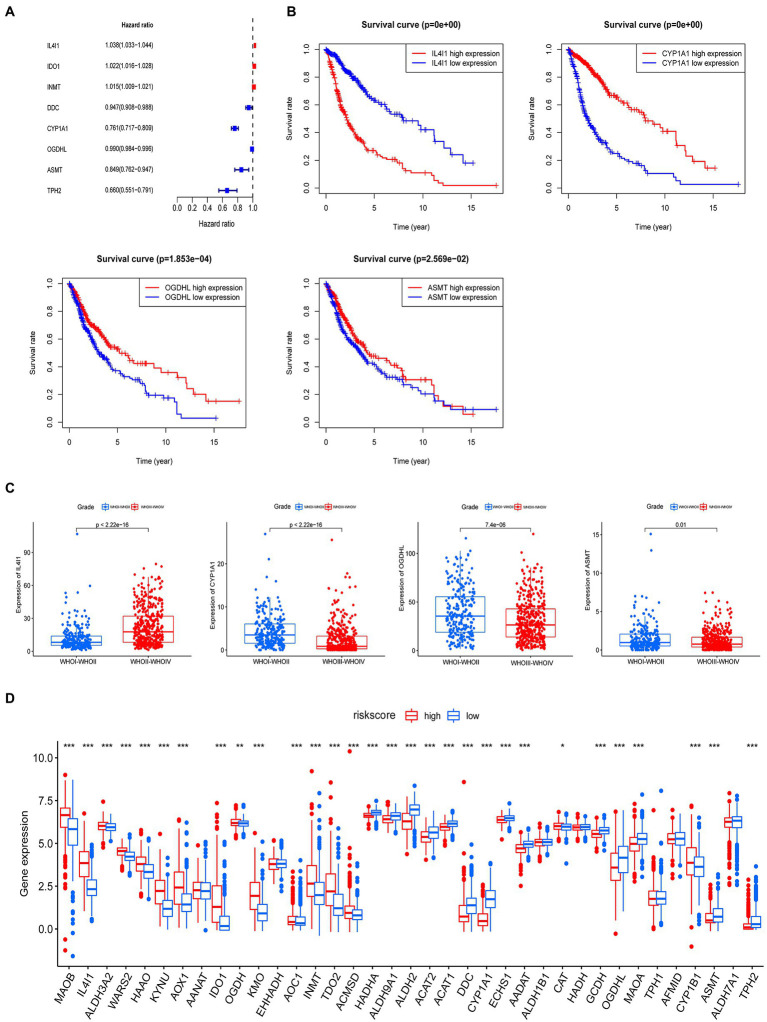
A prognostic model for predicting patients with glioma based on tryptophan metabolic gene expression. **(A)** Univariate Cox regression analysis demonstrated eight tryptophan metabolic genes were related to the prognosis of glioma. **(B)** Multivariate Cox regression analysis demonstrated four tryptophan metabolism genes were most relevant to the prognosis of glioma; a Kaplan–Meier curve for the overall survival of these genes (*IL4I1*, *CYPIA1*, *OGDHL*, and *ASMT*) is shown. **(C)** The relationship between *IL4I1*, *CYPIA1*, *OGDHL*, and *ASMT* and tumor stage. **(D)** The expression of 40 tryptophan metabolism genes in high and low risk groups. *p < 0.05, **p < 0.01, ***p < 0.001.

To fully understand the association between tryptophan metabolic gene expression patterns and tumorigenesis, 966 patients were collected from two eligible glioma cohorts (TCGA-LGG/GBM and CGGA) for further analysis. The details of 966 patients with gliomas are shown in Supplementary Table S1. Differentially expressed tryptophan metabolic genes between tumor and normal tissues were screened using |log fold change (FC)| ≥ 1 and *p* < 0.05. In total, nine differentially expressed genes were identified (Supplementary Table S2). We then performed univariate Cox regression and multivariate Cox regression analyses, in which four prognostic genes (*IL4I1*, *CYP1A1*, *OGDHL*, and *ASMT*) were found to be independent predictors (Table 1). Kaplan–Meier analysis showed that higher expression levels of *IL4I1* were associated with worse OS, while higher expression levels of *CYP1A1*, *OGDHL*, and *ASMT* were associated with better OS (Figure 4B). In addition, the expression levels of *IL4I1* in those with a World Health Organization tumor stage of 3–4 were significantly higher than that in those with a tumor stage of 1–2, while the expression levels of *CYP1A1*, *OGDHL,* and *ASMT* in those with a tumor stage of 3–4 were significantly lower than those with a tumor stage of 1–2 (Figure 4C). Combined with the results of our univariate Cox analysis (Figure 4A), we can conclude that *IL4I1* plays a tumor-promoting role and *CYP1A1*, *OGDHL*, and *ASMT* all play protective roles in gliomas.

**Table 1:** Multivariate Cox regression analysis of four tryptophan metabolic prognostic genes associated with overall survival in glioma patients.

Gene	Coef	HR	*p* value
*IL4I1*	0.030653968	1.031128639	<0.001
*CYP1A1*	−0.216700471	0.805171105	<0.001
*OGDHL*	0.00663866	1.006660745	<0.001
*ASMT*	−0.196764275	0.821384231	0.02569

Coef, coefficient and HR, hazard ratio.

The four tryptophan metabolic genes that were most strongly related to prognosis (*IL4I1*, *CYP1A1*, *OGDHL*, and *ASMT*) were used to construct a risk prediction model. The risk score was calculated as follows: risk score = 0.030653968 × (expression of *IL4I1*) + (−0.216700471) × (expression of *CYP1A1*) + 0.00663866 × (expression of *OGDHL*) + (−0.196764275) × (expression of ASMT). According to the median risk score, glioma patients were divided into low and high risk subtypes in both the TCGA and CGGA cohorts. We then explored the difference in tryptophan metabolic genes between the high and low risk score groups; the results showed that the expression of *MAOB*, *IL4I1*, *ALDH3A2*, *WARS2*, *HAAO*, *HYNU*, *AOX1*, *ICD1*, *OGDH*, *KMO*, *AOC1*, *INMT*, *TDO2*, *ACMSD*, and *CYP1B1* was significantly higher in the high than the low risk insurance group. The expression of *HADHA*, *ALDH9A1*, *ALDH2*, *ACAT2*, *ALAT1*, *DDC*, *CYP1A1*, *ECHS1*, *AADAT*, *CAT*, *GCDH*, *OGDHL*, *MAOA*, *AFMID*, *ASMT*, *ALDH7A1*, and *TPH2* was significantly lower in the high than the low risk group (Figure 4D).

**Figure 5:**
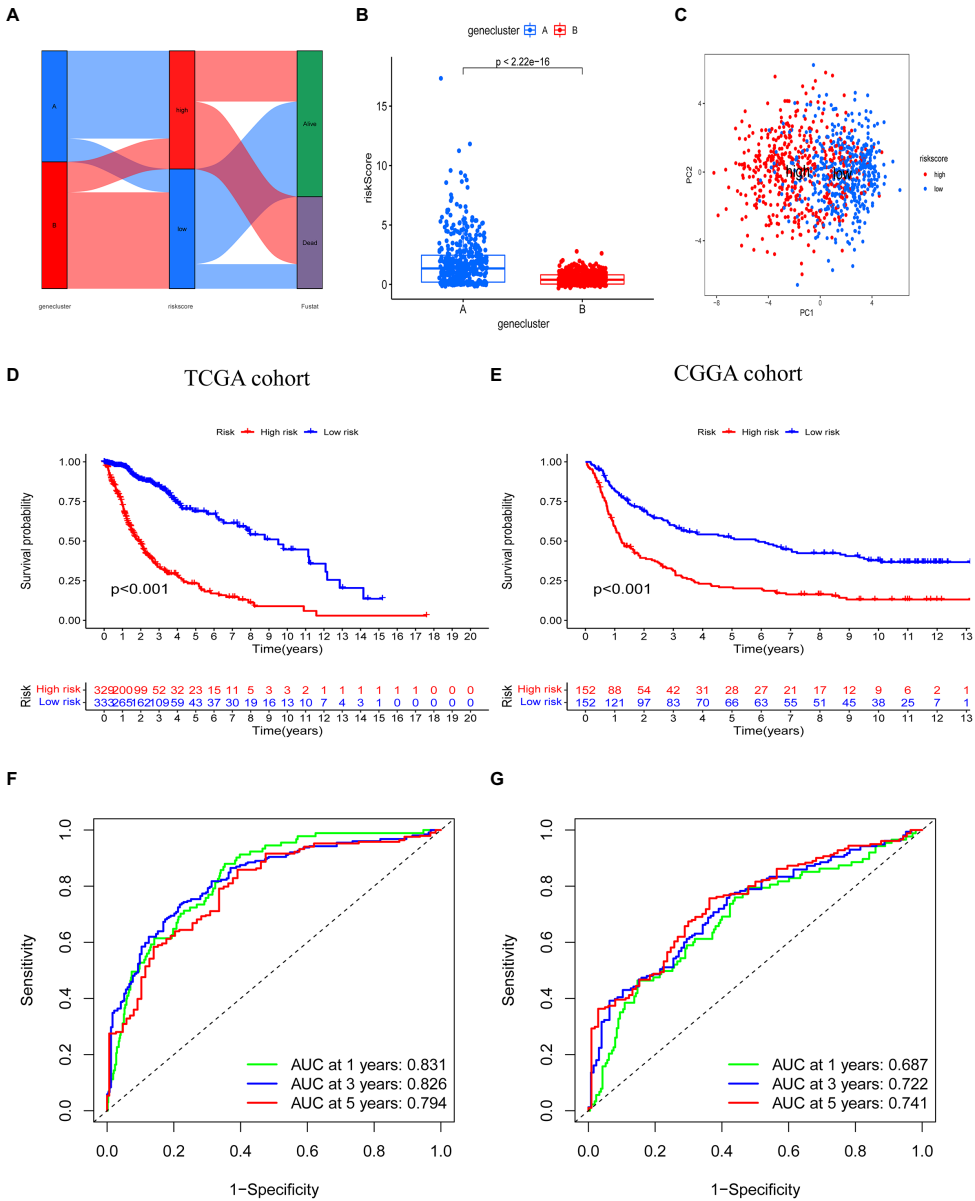
Prognostic value of the risk score model in glioma. **(A)** Alluvial diagram of subtype distributions in groups with different risk scores and survival outcomes. **(B)** Differences in risk score between genetic subtypes based on tryptophan metabolic gene expression. **(C)** Principal component analysis based on prognostic signature. High and low risk individuals are represented by red and blue dots, respectively. **(D,E)** Kaplan–Meier analysis of overall survival between the two groups in the TCGA and CGGA cohorts. **(F,G)** Receiver operating characteristic curves to predict the sensitivity and specificity of 1, 3, and 5-year survival according to risk score in TCGA and CGGA cohorts.

Figure 5A illustrates the distribution of glioma patients across the two subtypes (A vs. B), risk score groups (high vs. low), and patient survival outcome groups (dead vs. alive). We also analyzed the distribution of the two subtype groups in the risk score; the risk score for subtype A was significantly higher than subtype B. PCA analysis showed discernible differences between low risk and high risk groups (Figure 5C). The Kaplan–Meier survival curve confirmed that the OS of patients in the low risk group was significantly higher than in the high risk group in the TCGA cohort (Figure 5D, *p* < 0.001). In addition, the risk score model predicted 1, 3, and 5-year survival AUC values of 0.831, 0.826, and 0.794, respectively, in this cohort (Figure 5F). In the validation (CGGA) cohort, the OS of individuals in the low risk group was also significantly higher than that in the high risk group (Figure 5E, *p* < 0.001). In addition, the risk score model predicted 1, 3, and 5-year survival AUC values of 0.687, 0.722, and 0.741, respectively, in this cohort (Figure 5G).

### Analysis of prognostic risk score correlation with clinicopathological features

**Figure 6:**
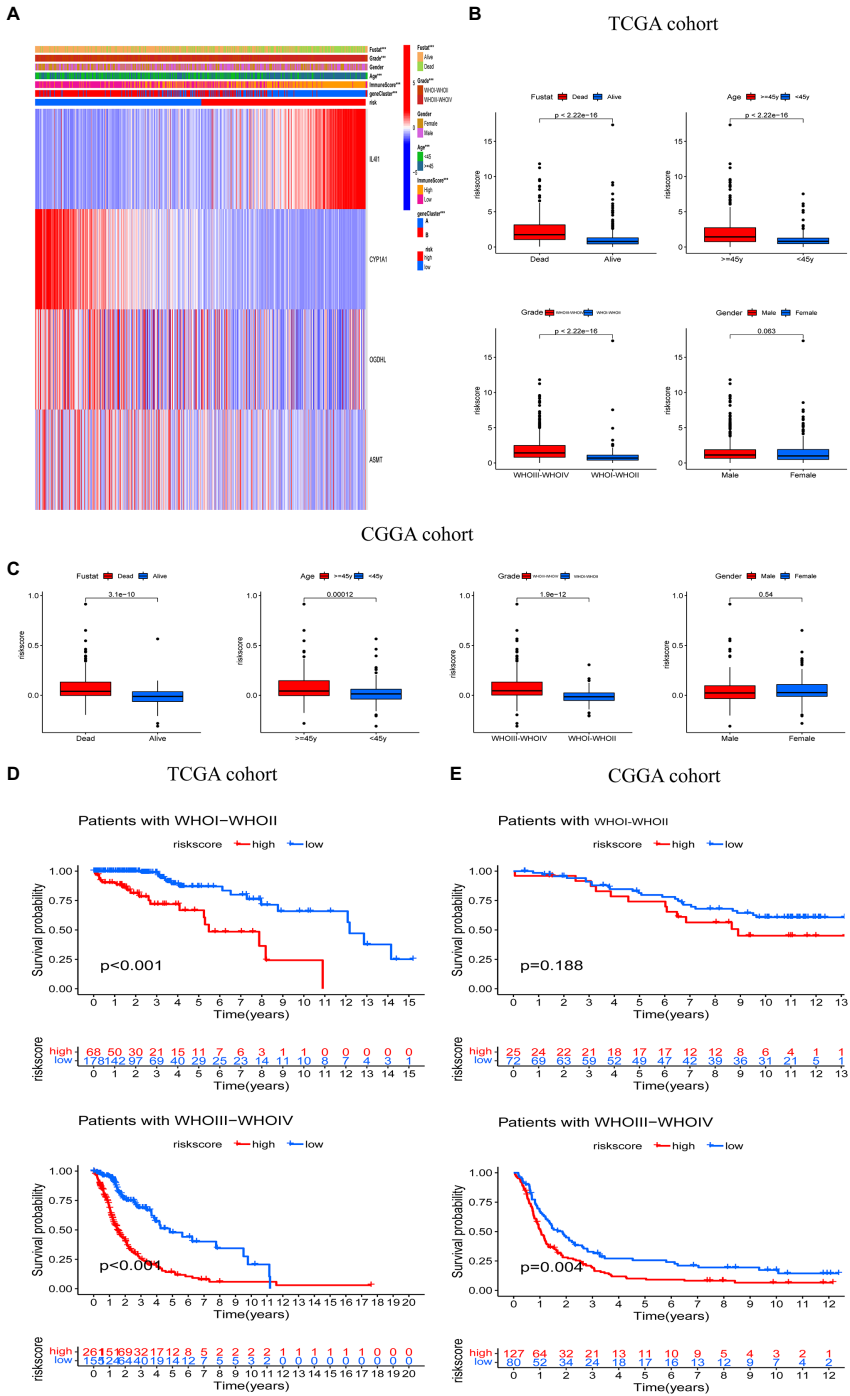
Correlation between risk score and clinical characteristics. **(A)** Relationship between clinicopathologic features and risk score. **(B,C)** Comparisons of overall survival (OS), WHO tumor stage, age, and gender between the two risk score groups in TCGA and CGGA cohorts. **(D,E)** Kaplan–Meier analysis of OS between the high and low risk groups in the TCGA and CGGA cohorts according to tumor stage. ***p < 0.001.

We then explored the relationship between risk score and clinicopathological features (Figure 6A). Risk scores were higher among those who died than those who survived; among those aged 45 years or older than those aged younger than 45 years; and among those with a tumor stage of 3–4 than those with a tumor stage of 1–2. However, there was no significant difference between genders in the TCGA cohort of the training set (Figure 6B). Similar results were observed in the CGGA validation set (Figure 6C). In addition, we analyzed OS among individuals with high and low risk scores and different tumor stages. The Kaplan–Meier survival curve showed that in the TCGA cohort, the OS of individuals in the high risk group was worse than those in the low risk score group, in those with a tumor stage of WHOI-WHOII or WHOIII-WHOIV (Figure 6D). In the CGGA cohort, among those with a tumor stage of WHOIII-WHOIV, the OS of patients in the high risk score group was worse than that of the low risk score group, but there was no difference in those with a tumor stage of WHOI-WHOII. However, overall, the prognosis of patients in the high risk score group was worse than that in the low risk score group (Figure 6E).

### Evaluation of TME, TMZ, and ICBs in high and low risk groups

**Figure 7:**
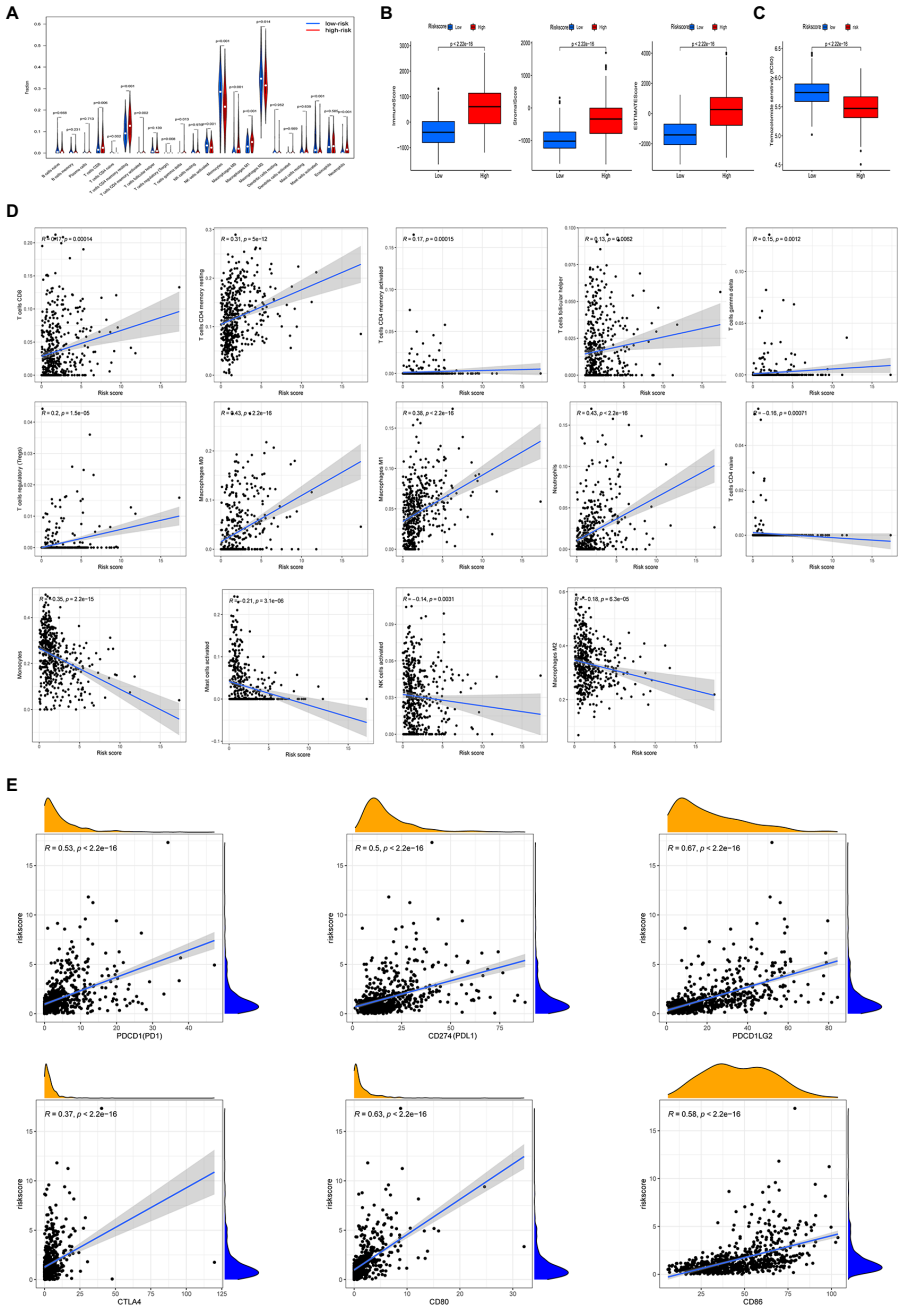
Different tumor microenvironment (TME) characteristics and response to temozolomide therapy between the two risk groups in TCGA cohort. **(A)** Abundance of tumor-infiltrating immune cells (TIICs) between high and low risk groups. **(B)** Comparisons of immune score, stromal score, and ESTIMATE score between high and low risk groups. **(C)** Half maximum inhibitory concentrations (IC50) for temozolomide between the two risk groups using data from the GDSC database. **(D)** Correlation between risk score and immune cell type abundance. **(E)** Correlation between risk score and immune checkpoint blockers (*PDCD1*, *CD274*, *PDCDLG2*, *CTLA4*, *CD80*, and *CD86*).

We used the CIBERSORT algorithm to evaluate the association between risk score and immune cell abundance. As shown in Figure 7A, the levels of infiltrating CD8^+^ T cells, activated CD4^+^ memory T cells, resting CD4^+^ memory T cells, regulatory T cells, γ δ T cells, M0 and M1 macrophages, and neutrophils were significantly higher in the high than the low risk group. The levels of infiltrating activated CD4^+^ T cells CD4, activated NK cells, monocytes, M2 macrophages, and activated mast cells were significantly higher in the low than the high risk group. Risk score was positively correlated with CD8^+^ T cells, activated CD4^+^ memory T cells, resting CD4^+^ memory T cells, follicular helper T cells, regulatory T cells, γ δ T cells, M0 and M1 macrophages, and neutrophils, and negatively correlated with activated CD4^+^ T cells, activated NK cells, monocytes, M2 macrophages, and activated mast cells (Figure 7D). The stromal, immune, and ESTIMATE scores were higher in the high than the low risk score group (Figure 7B). We also examined the relationship between immune checkpoint gene expression and risk score. The expression of the ICBs *PD1* and *CTLA* and their ligands (*PD-L1*, *PD-L2*, *CD80*, and *CD86*, all *p* < 0.001) were positively correlated with risk score (Figure 7E).

Considering that chemotherapy with temozolomide is a first-line treatment for gliomas, we investigated the response to temozolomide in people in the high and low risk groups based on data from the GDSC database. The log IC50 of temozolomide in those with the low risk subtype was significantly higher than that in patients with the high risk subtype. Therefore, the sensitivity to temozolomide in the high risk score group was higher than in the low risk score group (Figure 7C).

### The relationship between risk score and TMB

**Figure 8:**
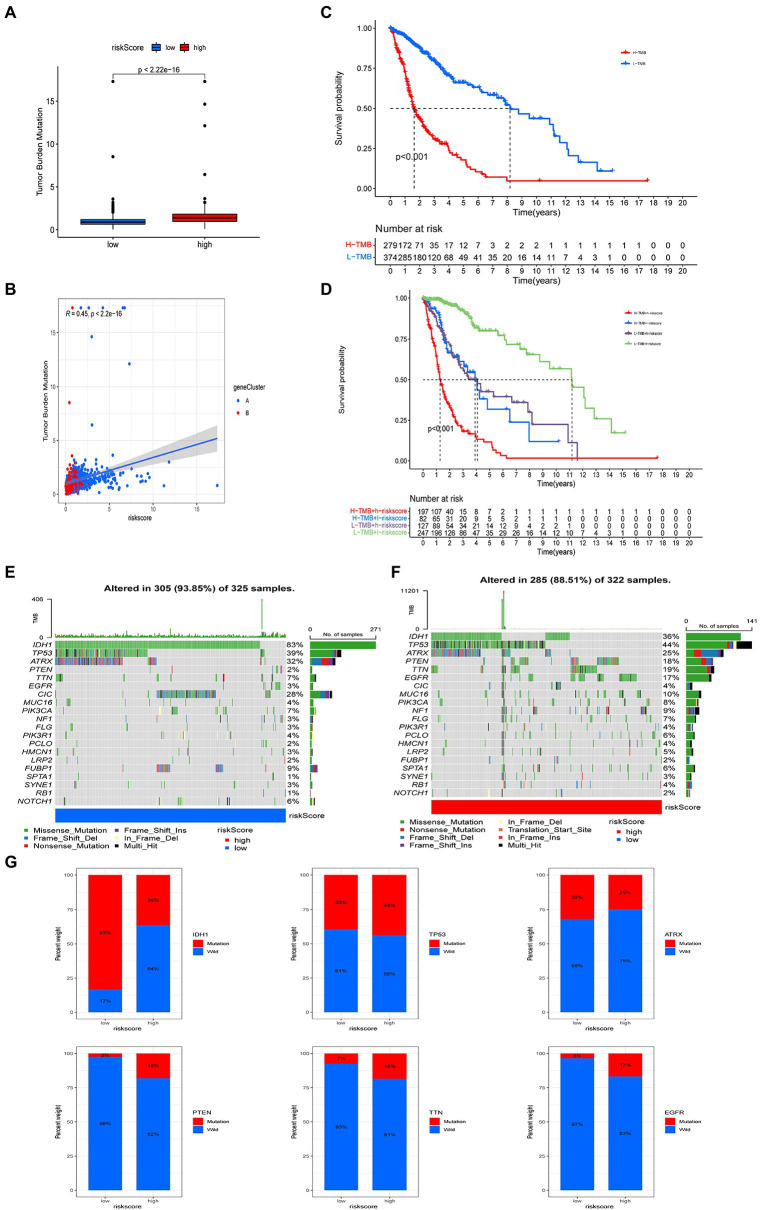
Comprehensive analysis of risk score in glioma. **(A)** Tumor mutational burden (TMB) in high and low risk score groups. **(B)** Kaplan–Meier analysis of overall survival (OS) between the high- and low-TMB groups. **(C)** Spearman’s correlation analysis of risk score and TMB. **(D)** Survival analysis among four groups stratified by risk score and TMB. **(E,F)** Waterfall plot of somatic mutation features associated with high and low risk scores. Each column represents one person. The upper bar plot shows TMB and the number on the right of the figure indicates mutation frequency for each gene. The right bar plot shows the proportion of each variant. **(G)** Comparisons of *IDH*, *TP53*, *EGFR*, *PTEN*, *TTN*, and *ATRX* mutations between high and low risk score groups.

Our analysis of TMB data from the TCGA-LGG/GBM queue shows that the TMB of the high risk score group is higher than that of the low risk score group (Figure 8A). Spearman’s correlation analysis showed that risk score was positively correlated with TMB (Figure 8B). We then analyzed the relationship between TMB and the overall survival rate among people with glioma, and the optimal cutoff, Kaplan–Meier survival curve showed that the OS of those in the high-TMB group was worse than that of those in the low-TMB group (Figure 8C). We then analyzed the relationship between risk, TMB, and OS among people with glioma. The Kaplan–Meier survival curve showed that the OS of patients in the H-TMB + H-risk score group was the worst, while that of L-TMB + L-risk score group was the best (Figure 8D).

We also analyzed the differences in somatic mutation distribution between high and low risk score groups in the TCGA cohort. The top 20 most mutated genes in the high and low risk score group were *IDH1*, *TP53*, *ATRX*, *PTEN*, *TTN*, *EGFR*, *CIC*, *MUC16*, *PIK3CA*, *NF1*, *FLG*, *PIK3R1*, *PCLO*, *HMCN1*, *LRP2*, *FUBP1*, *SPTA1*, *SYNE1*, *RB1*, and *NOTCH1* (Figures 8E,F). The frequencies of *IDH*, *TP53*, *ATRX*, and *CIC* mutations in patients with low risk scores were significantly higher than those with high risk scores. The opposite pattern was observed in the mutation levels of *PTEN*, *EGFR*, *TTN*, and *MUC16* (Figures 8E,F). The rates of *PTEN* (18%), *TTN* (19%), *EGFR* (21%), and *EGFR* (17%) mutations in patients with high risk scores were significantly different from those in patients with low risk scores (all *p* < 0.001). There was no significant difference in the frequency of *TP53* and *ATRX* mutations between the two groups (Figure 8G).

### The development of a nomograph for predicting OS

**Figure 9:**
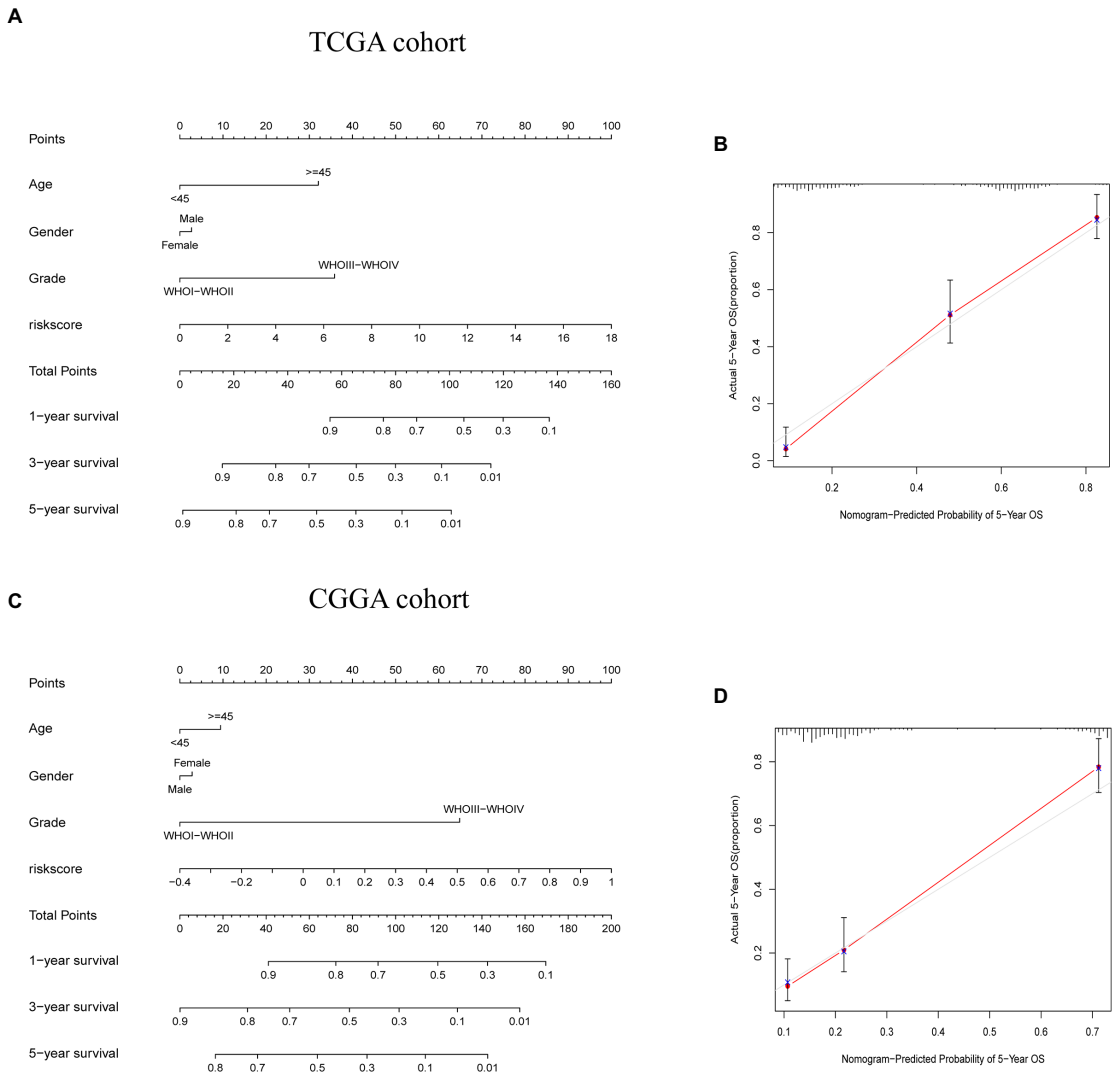
Construction and validation of a nomogram to predict overall survival (OS). **(A,C)** Nomogram for predicting 1, 3, and 5-year OS for people with glioma in the TCGA and CGGA cohorts. **(B,D)** Calibration curves for the nomogram to predict 5-year OS in TCGA and CGGA cohorts.

Considering the level of inconvenience associated with the clinical application of the risk score model in predicting OS in glioma patients, we established a nomogram incorporating risk score and clinicopathological parameters to predict OS survival rate at 1, 3, and 5 years among glioma patients (Figures 9A,C). Predictors included risk score, age, gender, and tumor stage. The calibration curve over 5 years shows that the proposed nomogram had a similar performance on both the training set and the test set compared with the ideal model (Figures 9B,D).

### Expression of the marker gene in the validation set

Finally, we also verified the expression of marker genes in the GSE4290 dataset. We discovered that the expression trends of IL4I1, CYP1A1, OGDHL, and ASMT were consistent with the TCGA dataset. Among them, the expression of IL4I1 in glioma patients was greater than of normal samples, while the CYP1A1, OGDHL, and ASMT were lower in glioma samples (Supplementary Figure S3A). In addition, we also verified the expression of marker genes in the GSE15824 dataset. We found that IL4I1 was highly expressed in glioma cell lines (LN018, LN215, LN229, and LN319), CYP1A1 was low expressed in glioma cell lines (LN215, LN229, LN319, and BS149), OGDHL was low expressed in LN018, and ASMT was lowly expressed in glioma cell lines (LN018, LN215, LN229, LN319, and BS149; Supplementary Figure S3B).

## Discussion

Many studies have shown that tryptophan metabolism plays an important role in regulating immunity and tumorigenesis (Kwiatkowska et al., 2021; Tanaka et al., 2021). However, most studies have focused on a single tryptophan metabolism gene or a single type of TME cell. Therefore, the combined effect of multiple tryptophan metabolic genes and their association with TME permeation characteristics have not been fully elucidated. The results of this study revealed genetic and transcriptional heterogeneity in tryptophan metabolic genes in gliomas. We identified two different molecular subtypes based on the expression of 40 tryptophan metabolic genes. Compared with patients with subtype B, patients with subtype A had worse clinicopathological features and OS. There were also significant differences in TME characteristics between the two subtypes in terms of cellular composition. In addition, the expression of different immune checkpoints in subtype A was higher than that in subtype B. Therefore, our results show that tryptophan metabolic genotyping could be used as a predictive index to evaluate the clinical outcome and immunotherapeutic response of gliomas. Therefore, we built a robust and effective prediction risk scoring model and verified its predictive ability. The construction of our predictive model was based on the expression levels of four genes (*IL4I1*, *CYP1A1*, *OGDHL*, and *ASMT*) in glioma tissues. The tryptophan metabolic subtypes characterized by immune activation and immunosuppression showed high risk score group and low risk score group, respectively. There were significant differences in clinicopathological features, prognosis, genetic mutation, TME, and immune checkpoints between the high and low risk score groups. Finally, a quantitative nomogram was established, in which risk score and clinicopathological features were incorporated, which further improved predictive performance and facilitated the use of the risk score prediction model. The prognostic model could be used to predict the prognosis of individuals with glioma and will provide novel ideas for targeted therapy.

The activation of the aromatic receptor AHR by tryptophan catabolites can enhance malignancy of a tumor and inhibit anti-tumor immunity (Perez-Castro et al., 2021). IL4I1 (part of our risk model) is able to activate AHR through the production of indole metabolites and canine, and is associated with decreased survival rate, promotion of cancer cell movement, and inhibition of adaptive immunity in people with glioma. However, IL4I1 could thus represent a new target for the treatment of glioma (Sadik et al., 2020). This is consistent with our study, which showed that IL4I1 plays a tumor-promoting role in gliomas. The human cytochrome P450 (CYP) 1A1 gene encodes a monooxygenase that can metabolize a variety of exogenous and endogenous substrates. CYP1A1 expression is mainly controlled *via* aromatic hydrocarbon receptors (AHR; Ma, 2001). Previous studies have shown that CYP1A1 is a promising target molecule in the prevention and treatment of human malignant tumors (Mescher and Haarmann-Stemmann, 2018). Dai and colleagues previously showed that low expression of the OGDHL enzyme is significantly associated with poor survival in patients with HCC. OGDHL is also a promising prognostic biomarker and a potential therapeutic target for OGDHL-negative liver cancer (Dai et al., 2020). However, it has not been studied in the context of glioma. In addition, previous studies have found that ASMT can regulate the invasiveness of breast cancer cells, and may be a potential drug target in breast cancer (Xie et al., 2020). However, there are few published studies on prognostic indicators and therapeutic targets in glioma, so our results reveal a novel role for four genes (*IL4I1, CYP1A1, OGDHL*, and *ASMT*) in this context, and that a predictive model that incorporates these genes is able to predict the prognosis of individuals with glioma. At the same time, it also provides novel potential therapeutic targets for the treatment of glioma.

In addition, in our study, the infiltration of CD8^+^ T cells, activated CD4^+^ memory T cells, resting CD4^+^ memory T cells, regulatory T cells, γ δ T cells, M0 and M1 macrophages, and neutrophils was upregulated in patients with high risk scores, while the infiltration of activated CD4^+^ T cells, activated NK cells, monocytes, M2 macrophages, and activate mast cells was downregulated in these individuals. Immunosuppression and low immune function are two important functional features of T regulatory cells (Wolf et al., 2015). Increased activity of rate-limiting enzymes such as IDO leads to the continuous consumption of tryptophan in the microenvironment. This leads to the stagnation of the cell cycle in surrounding T cells and promotes the production of T regulatory cells (Fallarino et al., 2006). Some studies have also shown that KYN activates AHR on CD4^+^ T cells *via* classical response genes such as *CYP1A1* and *CYP1B1*, thus inducing CD4^+^ T cell to differentiation into T regulatory cells (Mezrich et al., 2010). Therefore, T regulatory cells inhibit the function of effector T cells and regulate immune function by secreting inhibitory cytokines or interacting with antigen-presenting cells. In addition, AHR inhibits the function of CD8^+^ T cells by regulating the function of tumor-associated macrophages, resulting in anti-tumor immune responses (Takenaka et al., 2019). Our results also showed that NK cell infiltration is low in high risk individuals; it is reported that increased tryptophan catabolism can induce apoptosis of NK cells and lead to tumor immune escape, which may represent a possible reason for this observation (Grohmann and Bronte, 2010). Therefore, tumor immune evasion may be more likely in people with glioma who have high risk scores, thus negatively affecting the success of immunotherapy in these individuals.

In recent years, ICBs have been widely used in tumor immunotherapy; this includes PD-1 and PD-L1, which are used as immunotherapeutic agents in many tumor types, including lung cancer, bladder cancer, renal cell carcinoma, melanoma, lymphoma, and leukemia (Mayor et al., 2016; Krishnamurthy and Jimeno, 2017; Kaplon and Reichert, 2018). However, in a phase 3 clinical trial of glioblastoma, the effect of immunotherapy was found to be unsatisfactory (Weller et al., 2017; Reardon et al., 2020). Previous studies have found that PD-1 and PD-L1 can inhibit T cell proliferation in glioma (Litak et al., 2019), and studies in GBM mice have confirmed the safety and effectiveness of monoclonal antibodies against PD-1 and PD-L1, indicating that they have a high anticancer potential (Huang et al., 2015). Our results showed that there was a positive correlation between risk score and the expression of *PDCD1*, *CD274*, *PDCDLG2*, *CTLA4*, *CD80*, and *CD86*. In addition, we found that risk score was significantly associated with TMB, including several common somatic mutations (*TP53*, *PTEN*, and *EGFR*) in glioma (Segura-Collar et al., 2020). Risk score therefore plays an important role in the formation of the TME immune landscape, suggesting that the risk score model may affect the therapeutic effect of ICBs. Therefore, we concluded that the risk score model may be used to predict prognosis and immunotherapeutic response in patients with glioma.

This study has several limitations. First, all the analyses are based on the data from public databases, and all samples used in our study were therefore acquired retrospectively. Thus, an inherent bias in case selection may have affected the results. Large-scale prospective studies and further *in vivo* and *in vitro* studies are needed to confirm our findings. In addition, data on some important clinical variables, such as surgery, neoadjuvant chemotherapy, and radiotherapy, could not be analyzed in most data sets, which may affect immune responses and tryptophan metabolism.

## Conclusion

Our comprehensive analysis of tryptophan metabolic genes reveals a wide range of regulatory mechanisms by which the TME, clinicopathological features, and prognosis of glioma may be affected. Our results also indicate an association between tryptophan metabolic genes, immune checkpoints, and immunotherapy. These findings highlight the clinical significance of tryptophan metabolic genes and provide novel avenues for personalized immunotherapeutic strategies for people with glioma.

## Data availability statement

The original contributions presented in the study are included in the article/Supplementary material, further inquiries can be directed to the corresponding authors.

## Ethics statement

Ethical review and approval was not required for the study on human participants in accordance with the local legislation and institutional requirements. Written informed consent for participation was not required for this study in accordance with the national legislation and the institutional requirements.

## Author contributions

YL, CS, and GP conceived the study and collected and analyzed the data. YL and JX wrote the manuscript. GP provided technical guidance. YL, JX, YL, GP, and CS contributed to data collection, analysis and interpretation, and manuscript writing. All authors contributed to the article and approved the submitted version.

## Funding

This study was supported by the National Natural Science Foundation of China (no. 81801908), the Hunan Provincial Natural Science Committee Project (no. 2021JJ70074 and 2022JJ70163), and Hunan Province Science and Technology Major Project (no. 2019SK1010).

## Conflict of interest

The authors declare that the research was conducted in the absence of any commercial or financial relationships that could be construed as a potential conflict of interest.

## Publisher’s note

All claims expressed in this article are solely those of the authors and do not necessarily represent those of their affiliated organizations, or those of the publisher, the editors and the reviewers. Any product that may be evaluated in this article, or claim that may be made by its manufacturer, is not guaranteed or endorsed by the publisher.

## Supplementary material

The Supplementary material for this article can be found online at: https://www.frontiersin.org/articles/10.3389/fnmol.2022.1037835/full#supplementary-material

Supplementary Figure S1 Mutational frequencies of 40 tryptophan metabolic genes in 390 and 506 patients with **(A)** GBM and **(B)** LGG, from the TCGA cohort.

Supplementary Figure S2 Classification of patients according to tryptophan metabolic gene expression profile. **(A,B)** Consensus clustering cumulative distribution function (CDF) for k=2 to k=9. Relative change in the area under the CDF curve for k=2 to k=9.

Supplementary Figure S3 Expression of the marker gene (IL4I1,CYP1A1,OGDHL and ASMT) in the validation set. **(A)** The expression of marker genes in the GSE4290 dataset. **(B)** The expression of marker genes in the GSE15824 dataset.

## Footnotes

^1^ https://xenabrowser.net/datapages/

^2^ http://www.broad.mit.edu/gsea/msigdb/

## Abbreviations

TME: Tumor microenvironment
ICBs: Immune checkpoint blockers
OS: Overall survival
TTFields: Tumor electric field therapy
TMZ: Temozolomide
IDO1: Indoleamine-2,3-dioxygenase-1
IDO2: Indoleamine-2,3-dioxygenase-2
TIIC: Tumor-infiltrating immune cells
GTEx: Genotype–Tissue Expression
CGGA: Chinese Glioma Genome Atlas
TCGA: The Cancer Genome Atlas
TPM: Transcripts per kilobase million
CDF: Cumulative distribution function
TMB: Tumor mutational burden
CNV: Copy number variation
PCA: Principal component analysis
ROC: Receiver operating characteristic
GDSC: Genomics of Drug Sensitivity in Cancer
HCC: Hepatocellular carcinoma
