# Supplementary figures and images for: Identification of tryptophan metabolic gene-related subtypes, development of prognostic models, and characterization of tumor microenvironment infiltration in gliomas
Source: Front Mol Neurosci. 2022 Nov 4;15:1037835. doi: 10.3389/fnmol.2022.1037835 (PMC9673907; doi:10.3389/fnmol.2022.1037835)

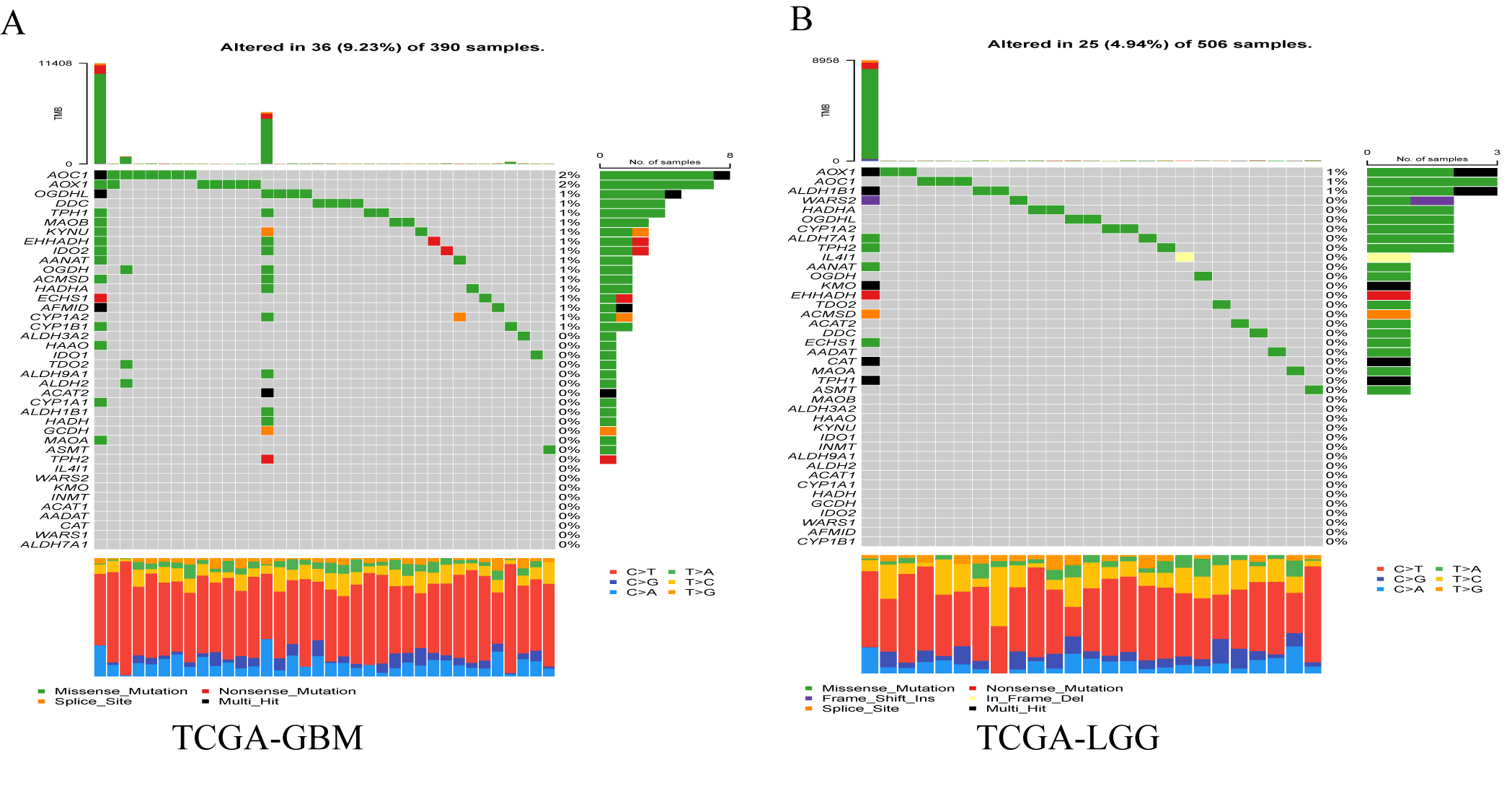

Supplement: Supplementary Figure S1 — Mutational frequencies of 40 tryptophan metabolic genes in 390 and 506 patients with (A) GBM and (B) LGG, from the TCGA cohort. [file Figure_S1.TIF]

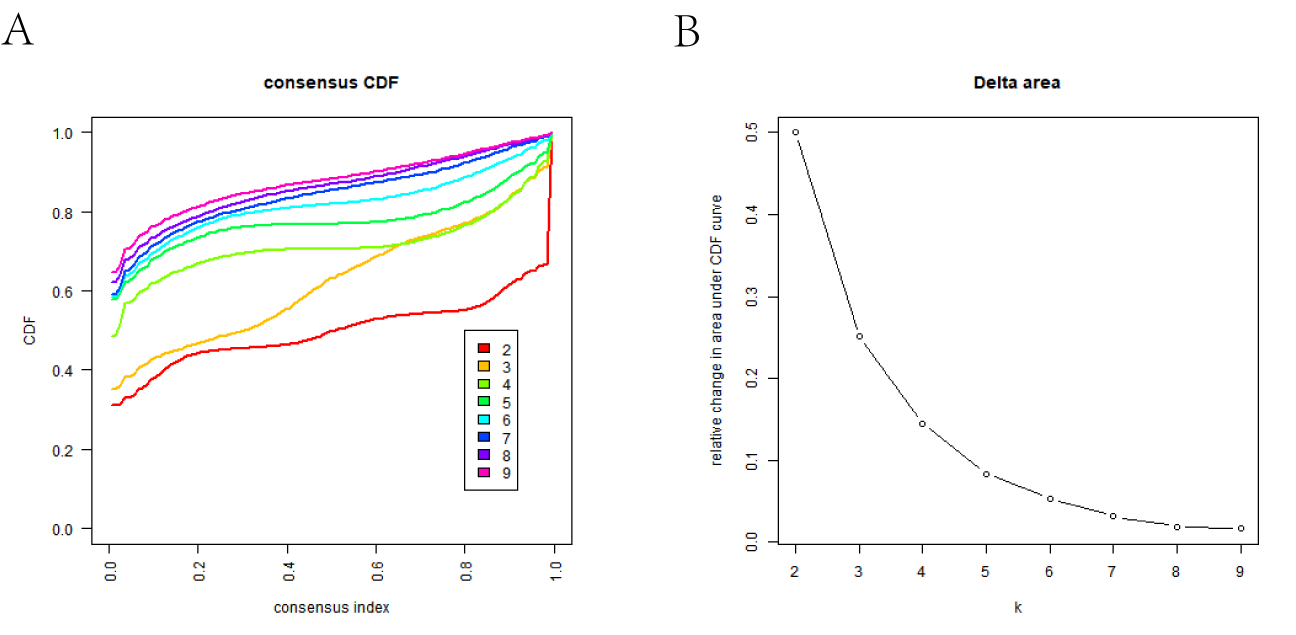

Supplement: Supplementary Figure S2 — Classification of patients according to tryptophan metabolic gene expression profile. (A,B) Consensus clustering cumulative distribution function (CDF) for k=2 to k=9. Relative change in the area under the CDF curve for k=2 to k=9. [file Figure_S2.TIF]

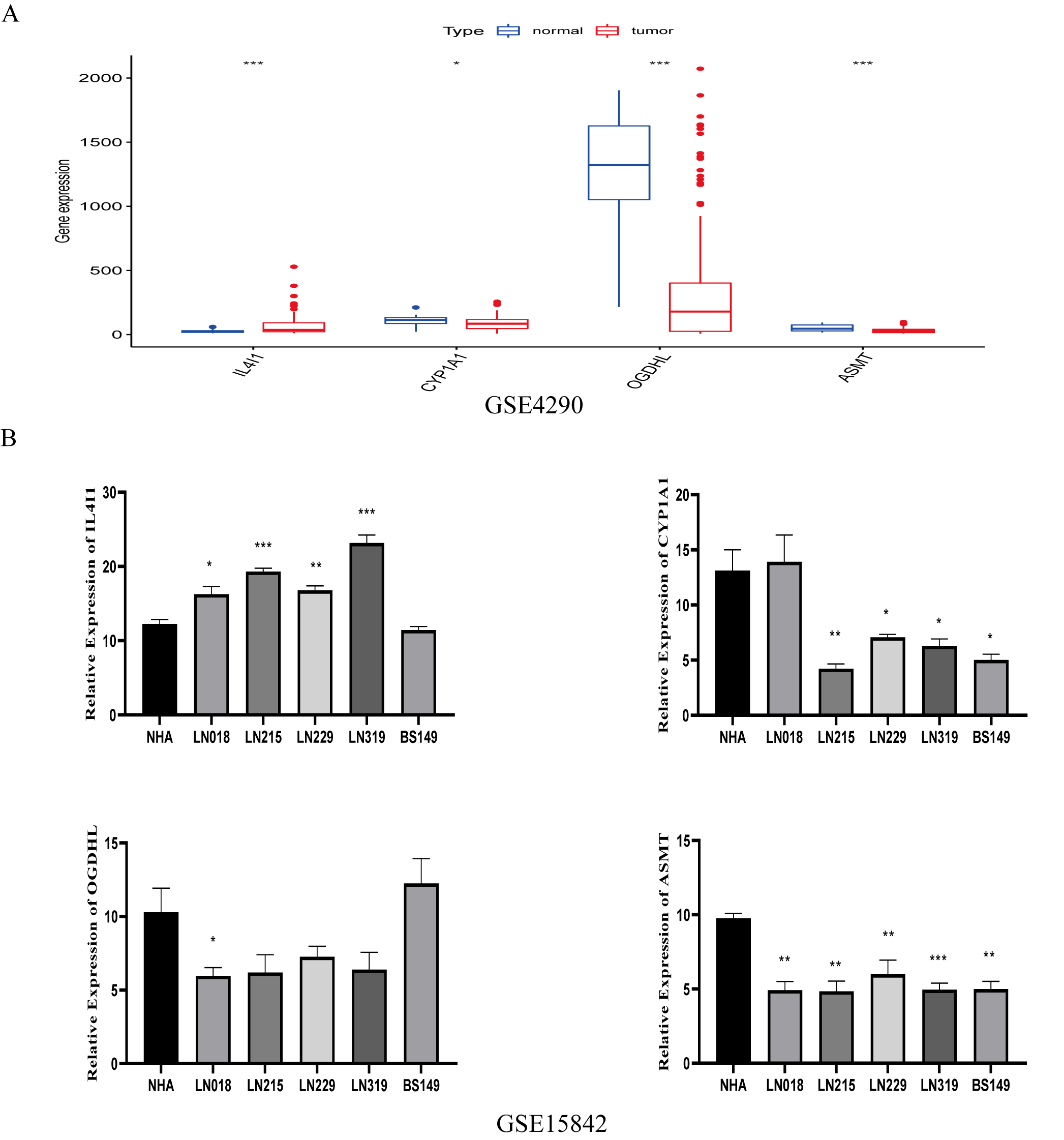

Supplement: Supplementary Figure S3 — Expression of the marker gene (IL4I1,CYP1A1,OGDHL and ASMT) in the validation set. (A) The expression of marker genes in the GSE4290 dataset. (B) The expression of marker genes in the GSE15824 dataset. [file Figure_S3.TIF]
